# Identification of cancer risk and associated behaviour: implications for social marketing campaigns for cancer prevention

**DOI:** 10.1186/s12885-017-3540-x

**Published:** 2017-08-17

**Authors:** Rebecca Kippen, Erica James, Bernadette Ward, Penny Buykx, Ardel Shamsullah, Wendy Watson, Kathy Chapman

**Affiliations:** 1 School of Rural Health, Monash University, PO Box 666, Bendigo, VIC 3552 Australia; 2 School of Medicine and Public Health, University of Newcastle, University Drive, Callaghan, NSW 2308 Australia; 3 School of Health and Related Research, University of Sheffield, Regent Court, 30 Regent Street, Sheffield, S1 4DA UK; 4 Cancer Council New South Wales, PO Box 572, Kings Cross, Sydney, NSW 1340 Australia

**Keywords:** Cancer, Social marketing, Risk factors

## Abstract

**Background:**

Community misconception of what causes cancer is an important consideration when devising communication strategies around cancer prevention, while those initiating social marketing campaigns must decide whether to target the general population or to tailor messages for different audiences. This paper investigates the relationships between demographic characteristics, identification of selected cancer risk factors, and associated protective behaviours, to inform audience segmentation for cancer prevention social marketing.

**Methods:**

Data for this cross-sectional study (*n* = 3301) are derived from Cancer Council New South Wales’ 2013 Cancer Prevention Survey. Descriptive statistics and logistic regression models were used to investigate the relationship between respondent demographic characteristics and identification of each of seven cancer risk factors; demographic characteristics and practice of the seven ‘protective’ behaviours associated with the seven cancer risk factors; and identification of cancer risk factors and practising the associated protective behaviours, controlling for demographic characteristics.

**Results:**

More than 90% of respondents across demographic groups identified sun exposure and smoking cigarettes as moderate or large cancer risk factors. Around 80% identified passive smoking as a moderate/large risk factor, and 40–60% identified being overweight or obese, drinking alcohol, not eating enough vegetables and not eating enough fruit. Women and older respondents were more likely to identify most cancer risk factors as moderate/large, and to practise associated protective behaviours. Education was correlated with identification of smoking as a moderate/large cancer risk factor, and with four of the seven protective behaviours. Location (metropolitan/regional) and country of birth (Australia/other) were weak predictors of identification and of protective behaviours. Identification of a cancer risk factor as moderate/large was a significant predictor for five out of seven associated cancer-protective behaviours, controlling for demographic characteristics.

**Conclusions:**

These findings suggest a role for both audience segmentation and whole-of-population approaches in cancer-prevention social marketing campaigns. Targeted campaigns can address beliefs of younger people and men about cancer risk factors. Traditional population campaigns can enhance awareness of being overweight, alcohol consumption, and poor vegetable and fruit intake as cancer risk factors.

**Electronic supplementary material:**

The online version of this article (doi:10.1186/s12885-017-3540-x) contains supplementary material, which is available to authorized users.

## Background

In Australia, around one-third of cancer cases are due to six modifiable lifestyle risks: tobacco use, ultraviolet radiation exposure, inadequate diet, overweight and obesity, alcohol consumption, and lack of physical activity [1]. This reflects international research that finds globally, around one-third of cancer deaths are due to smoking, low fruit and vegetable intake, overweight and obesity, alcohol consumption, and physical inactivity [2].

One obstacle that challenges cancer prevention is the existing community scepticism that cancer can be prevented, [3] despite research indicating that only 5% of cancers are hereditary [3, 4]. There is currently poor community understanding of what causes cancer and how to reduce risk [5, 6]. In an Australian study examining cancer patient beliefs surrounding the development of cancer, almost half of respondents did not know what factors contributed to their cancer, whilst few participants identified behavioural risks as causal factors [5]. Similarly, in a study investigating the beliefs and perceptions held by women regarding the causes of breast cancer, results indicated that while most respondents nominated factors that they believed contribute to the development of breast cancer, a large number of these factors are not supported by scientific evidence. The authors conclude that such misunderstandings of the causes of cancer could negatively affect the efficacy of campaigns for cancer prevention [6].

Compared to tobacco and sun exposure, there is much lower recognition of the carcinogenic effects of the other main established lifestyle causes of cancer, especially obesity, physical inactivity and alcohol [7–9]. Sun exposure and diet show significant recognition in some national surveys but not in others. One UK study that examined public awareness of the associations with lifestyle of both cancer and heart disease found that awareness was significantly higher for the latter, with the authors noting that the link between lifestyle factors and cancer (bar lung cancer) has been publicised only recently [7]. In Australia, results from a series of surveys largely parallel international findings, indicating high awareness of the links between smoking and lung cancer (96%) and sun exposure and melanoma (80%), but much lower, although marginally improving, awareness of other health-behaviour cancer risk factors [8]. The 2000 survey in this series found that fewer than 40% of people believed that they could ‘greatly reduce’ their personal risk of cancer. Awareness of lifestyle factors associated with cancer were lower than for other common diseases [8]. Knowledge of the cancer risks of too much alcohol increased from 41% in 2006 to 48% in 2009, however remained well below awareness that excessive drinking could result in liver problems (98%) or being overweight or obese (89%) [9].

Community perceptions of avoidable cancer risk factors are a vital consideration in the development of communication strategies for cancer prevention [5]. Public awareness of cancer risk is of significant importance if messages about changing behaviour and engaging in screening are to be seen as relevant by target groups. Whilst it is unrealistic to expect that public health campaigns could utterly transform entrenched unhealthy lifestyles, they can make a difference when their messages reach and are absorbed by the public. Even modest improvements in health behaviours, given the scale of the problem of unhealthy lifestyles, can make a substantial improvement to population mortality and morbidity. International studies of public awareness of cancer risks consistently show that the primary problem is that many people have only patchy appreciation of the gravity of cancer risks associated with what may seem ‘normal’ lifestyles [6–8]. Multicomponent, comprehensive health promotion is most likely to effectively influence behaviour change [10] and this may include social marketing campaigns.

*Social marketing* applies commercial marketing strategies to modify social behaviours for the benefit of the community, including those behaviours related to public health [11]. Methods are “drawn from behavioural theory, persuasion psychology, and marketing science with regard to health behaviour, human reactions to messages and message delivery, and the ‘marketing mix’ or ‘four Ps’ of marketing (place, price, product, and promotion)” [11, 12]. As a discipline, modern social marketing has started to incorporate the powerful tool set utilised by commercial marketing professionals, such as analysing specific audiences and targeting them with customised messages [11]. Using mediums such as print media, television, radio, digital media and billboards, health-related social marketing campaigns are implemented with intent of effecting voluntary change in the health behaviours of large populations, often in part through provoking cognitive or emotional responses within audiences as a result [13, 14].

Those initiating social marketing campaigns must decide whether to target the general population or to tailor messages for different audiences with differing demographic, cultural, or behavioural characteristics [11]. Audience segmentation for cancer prevention may be particularly important since there are geographical and social differences in cancer awareness and related health outcomes. Social marketing therefore requires analysis of the relationship between demographic characteristics, current knowledge of cancer risk factors and current behaviours. This paper aims to investigate the relationship between respondent demographic characteristics and identification of seven cancer risk factors, and to determine whether respondents who identified a particular cancer risk factor as ‘Moderate/large’ were more likely to practise the recommended behaviour.

## Methods

Data for this study are from the Cancer Prevention Survey carried out in 2013 by Cancer Council New South Wales (NSW) [see Additional file 1]. There were 3301 respondents to the 20-min survey, all of whom were adult residents of NSW. Each respondent was randomised to three of four cancer-prevention topics: sun protection (*n* = 2474 respondents), tobacco control (*n* = 2473), nutrition (*n* = 2474) and alcohol (*n* = 2482) [15].

The sample was recruited by a market research company from their participant database. An invitation to respond to an online survey on personal health was emailed to 30,179 adults living in NSW. Of these, 5290 began the screening questions. A total of 962 people were screened out because quota limits for respondent age, sex, location and education had been reached (*n* = 760), because they were undergoing treatment for cancer (*n* = 123), or because they worked in the advertising industry or in the manufacture or sale of alcohol or tobacco products (*n* = 79). Another 983 began the survey but did not complete it, and 44 responses were omitted because they were completed in less than one-third of the median survey time or showed minimal variability across scale items [15].

### Demographic characteristics

Demographic characteristics of respondents included in this study were sex (male/female), age (18–29 years, 30–49 years, 50 years and over), residential location (metro/regional), education (up to year 10, year 11 or 12, diploma/certificate, university degree) and country of birth (Australia/other). These questions were answered by all 3301 respondents. Responses were weighted in the reported analyses so that the sample reflected the distribution of the NSW adult population by sex, age, location and education [15].

### Identification of cancer risk factors

Respondent identification of cancer risk factors was captured through the question ‘How much do each of the following things contribute to a person’s risk of getting cancer?’, with scale responses ‘None’, ‘Slight’, ‘Moderate’ and ‘Large’, and an alternative option of ‘Don’t know’.

The list of cancer risk factors included the following seven behavioural items (see also Table 1):

1. Spending time outdoors during peak ultraviolet radiation (UV) times without sun protection
2. Smoking cigarettes
3. Passive smoking
4. Being overweight or obese
5. Drinking alcohol
6. Not eating enough vegetables
7. Not eating enough fruit

**Table 1:** Seven cancer risk factors and associated risk and protective behaviours

Cancer risk factor	Risk behaviour	Protective behaviour
1. Spending time outdoors during peak UV times without sun protection	1. Tried to get a tan from the sun or used a solarium this summer	1. Sunsafe: Did not try to get a tan from the sun nor used a solarium this summer
2. Smoking cigarettes	2. Daily or occasional smoker of cigarettes, cigars or pipes	2. Non-smoker: Does not smoke cigarettes, cigars nor pipes
3. Passive smoking	3. Does not try to avoid places where s/he may be exposed to other people’s cigarette smoke	3. Avoid passive smoke: Tries to avoid places where s/he may be exposed to other people’s cigarette smoke
4. Being overweight or obese	4. Body Mass Index (BMI) of 25 or more	4. Healthy weight: BMI of 18.5 to less than 25.0
5. Drinking alcohol	5. AUDIT-C score of 4 or more for men and 3 or more for women	5. Lower risk alcohol intake: AUDIT-C score of 0–3 for men and 0–2 for women
6. Not eating enough vegetables	6. Eating fewer than five serves of vegetables per day	6. 5+ vegetable serves: Eating five or more serves of vegetables per day
7. Not eating enough fruit	7. Eating fewer than two serves of fruit per day	7. 2+ fruit serves: Eating two or more serves of fruit per day

### Behaviour

Each of these seven risk factors was matched with an associated behaviour, self-reported by the respondent in the survey, and coded as either a ‘protective’ or ‘risk’ behaviour. These are outlined below (see also Table 1).

#### 1. Risk factor: spending time outdoors during peak UV times without sun protection

Risk behaviour: Tried to get a tan from the sun or used a solarium this summer

This behaviour was measured by responses to two questions: ‘Which of the following things have you done this summer?’—‘Used a solarium’; and ‘Which of the following things have you done this summer?’—‘Tried to get a tan from the sun’. The survey was carried out at the end of the Australian summer in February 2013. Possible response categories were ‘Yes’, ‘No’ and ‘Don’t know’. Of the 2474 participants who answered these questions, 1973 (79.7%) stated ‘No’ to both, while 501 (20.3%) said ‘Yes’ or ‘Don’t know’ to one or both questions. ‘Don’t know’ constituted 0.7% of responses to ‘Tried to get a tan from the sun’ and 1.1% of responses to ‘Used a solarium’. Those who responded ‘No’ to both questions were coded as exhibiting protective ‘Sunsafe behaviour’ while the other 501 respondents were coded as ‘Sun vulnerable behaviour’.

#### 2. Risk factor: smoking cigarettes

Risk behaviour: Daily or occasional smoker of cigarettes, cigars or pipes

All survey participants were asked ‘Which of the following best describes your smoking status? This includes cigarettes, cigars and pipes.’ with possible responses ‘I smoke daily’, ‘I smoke occasionally’, ‘I don’t smoke now, but I used to’, ‘I’ve tried it a few times, but never smoked regularly’, and ‘I’ve never smoked’. The first two of these responses were coded as ‘Smoker’ (*n* = 567, 17.2%) with the last three coded as ‘Non-smoker’ (*n* = 2734, 82.8%).

#### 3. Risk factor: passive smoking

Risk behaviour: Does not try to avoid places where s/he may be exposed to other people’s cigarette smoke

This behaviour was coded from 2473 responses to the statement ‘I try to avoid places where I may be exposed to other people's cigarette smoke’. ‘Agree’ and ‘Strongly agree’ were coded as ‘Avoids passive smoke’ (*n* = 1681, 68.0%) while ‘Disagree’, ‘Strongly disagree’, ‘Neither’ and ‘Don’t know’ were coded as ‘Does not avoid passive smoke’ (*n* = 792, 32.0%).

#### 4. Risk factor: being overweight or obese

Risk indicator: Body Mass Index (BMI) of 25 or more

Height and weight were asked of all 3301 survey participants but reported by only 2790 respondents. BMI, equal to [weight in kilograms]/[(height in metres)^2^], was calculated for these respondents. Fifty respondents (1.8%) with BMIs of less than 18.5 (‘Underweight’) were excluded from analysis. Respondents with BMIs of 18.5 to less than 25.0 were coded as ‘Healthy weight’ (*n* = 1087, 39.0%). Respondents with BMIs of 25.0 or greater were coded as ‘Overweight’ (*n* = 1653, 59.2%). These BMI ranges for ‘Underweight’, ‘Healthy weight’ and ‘Overweight’ follow Australian Healthy Weight guidelines [16].

#### 5. Risk factor: drinking alcohol

Risk behaviour: AUDIT-C score of four or more for men and three or more for women

The AUDIT-C (Alcohol Use Disorders—Identification Test—Consumption) score measures alcohol consumption on a scale from 0 to 12 and is designed to detect heavy drinking or alcohol abuse [17]. Questions on personal alcohol consumption were asked of the 2482 survey participants randomised to the cancer-prevention topic around alcohol, and answered by 2462 respondents, allowing calculation of individual AUDIT-C scores. Following Rubinsky et al., [18] scores of 4 or more for men and 3 or more for women were coded as ‘Higher-risk alcohol intake’ (*n* = 1361, 55.3%). Scores of 0–3 for men and 0–2 for women were coded as ‘Lower-risk alcohol intake’ (*n* = 1101, 44.7%).

#### 6. Risk factor: not eating enough vegetables

Risk behaviour: Eating fewer than five serves of vegetables per day

There were 2474 answers to the question ‘If a serve of vegetables is equal to half a cup of cooked vegetables, one medium potato or one cup of salad, how many SERVES of vegetables do you eat each day, on average?’ with responses coded as ‘5 or more vegetable serves’ (*n* = 255, 10.3%) and ‘Less than 5 vegetable serves’ (*n* = 2219, 89.7%). This follows Australian government guidelines for recommended daily vegetable intake [19].

#### 7. Risk factor: not eating enough fruit

Risk behaviour: Eating fewer than two serves of fruit per day

This behaviour was coded from responses to ‘If a serve of fruit is equal to one medium piece or two small pieces of fruit, or one cup of diced fruit, how many SERVES of fruit do you eat each day, on average?’. Of 2474 respondents, 1417 (57.3%) were coded as ‘2 or more fruit serves’ and 1057 (42.7%) were coded as ‘Less than 2 fruit serves’. This follows Australian government guidelines for recommended daily fruit intake [19].

### Analysis

Seven logistic regressions were conducted to investigate the relationship between respondent demographic characteristics and identification of each of the seven cancer risk factors as either ‘Moderate/large’ or ‘None/slight’ (with ‘Don’t know’ responses excluded). A second set of logistic regressions was conducted to examine the relationship between respondent demographic characteristics and the seven ‘protective’ behaviours outlined in the previous section. Finally, a third set of regressions were conducted to determine whether respondents who identified a particular cancer risk factor as ‘Moderate/large’ were more likely to practise the associated ‘protective’ behaviour than were respondents who identified the risk factor as ‘None/slight’, controlling for demographic characteristics.

## Results

**Fig. 1:**
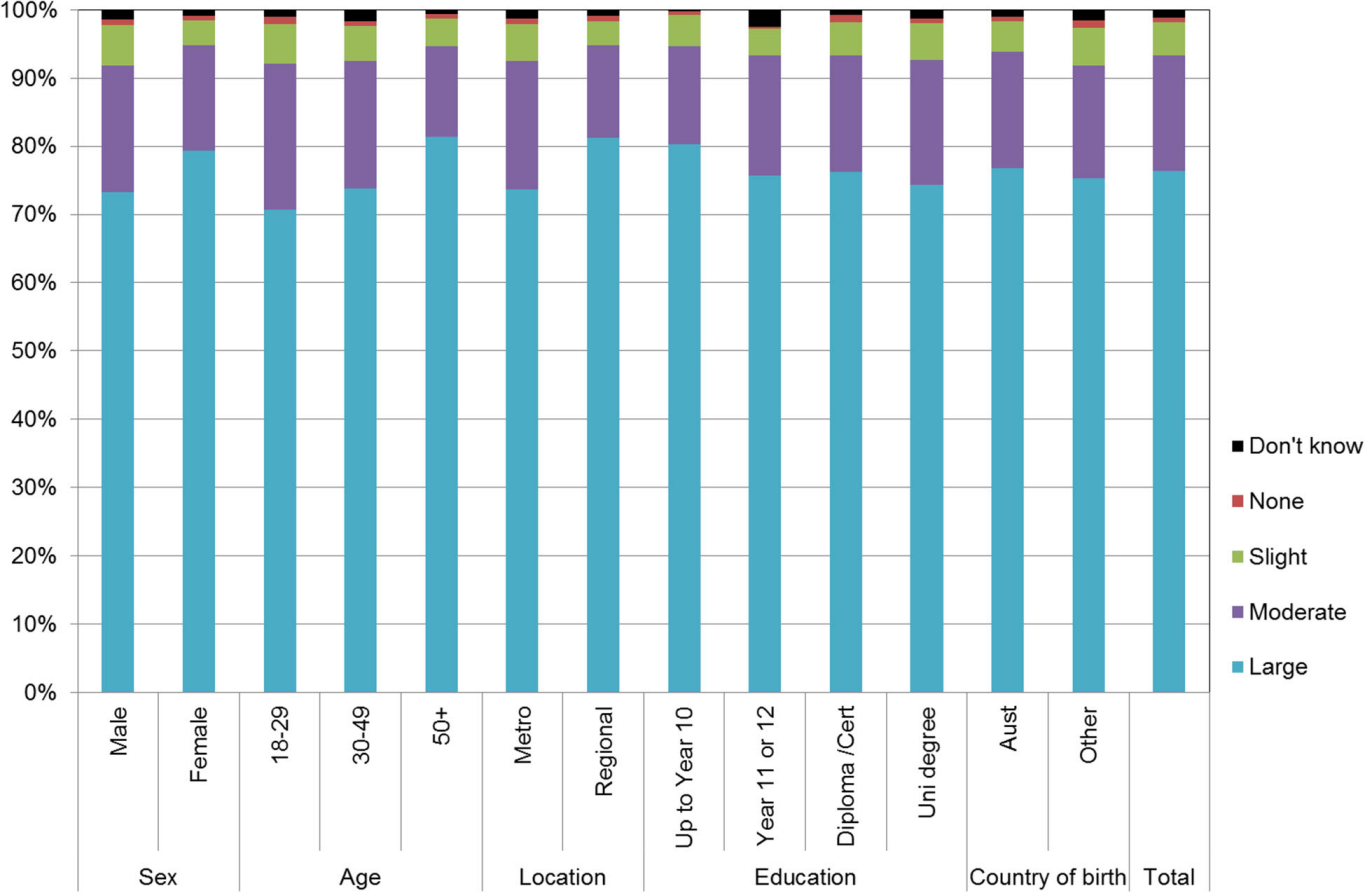
How much does spending time outdoors during peak UV times without sun protection contribute to a person’s risk of getting cancer? Responses by respondent demographic characteristics

**Fig. 2:**
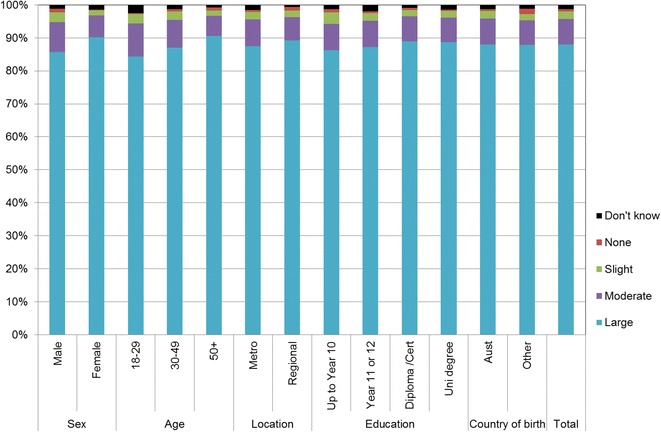
How much does smoking cigarettes contribute to a person’s risk of getting cancer? Responses by respondent demographic characteristics

**Fig. 3:**
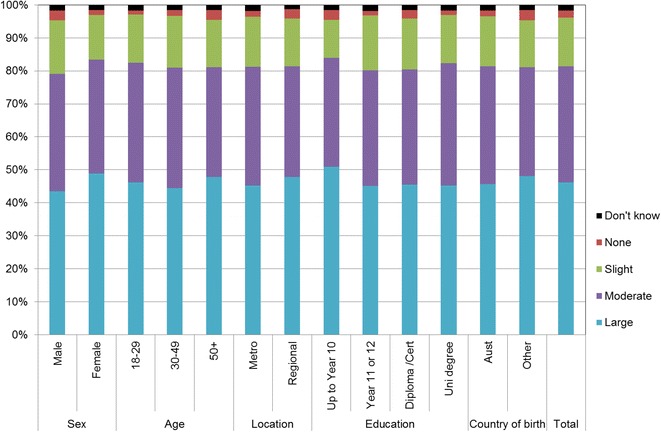
How much does passive smoking contribute to a person’s risk of getting cancer? Responses by respondent demographic characteristics

**Fig. 4:**
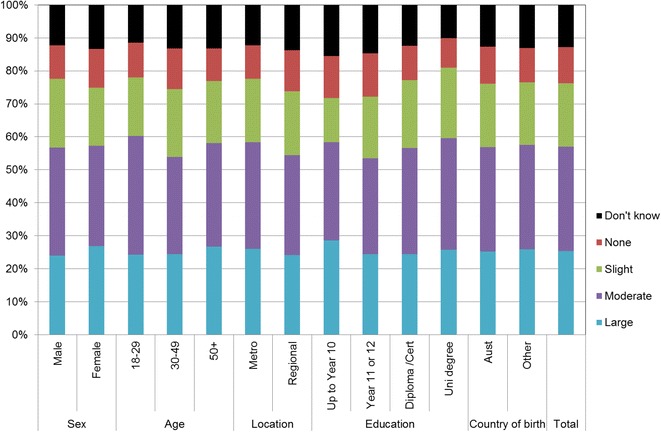
How much does being overweight contribute to a person’s risk of getting cancer? Responses by respondent demographic characteristics

**Fig. 5:**
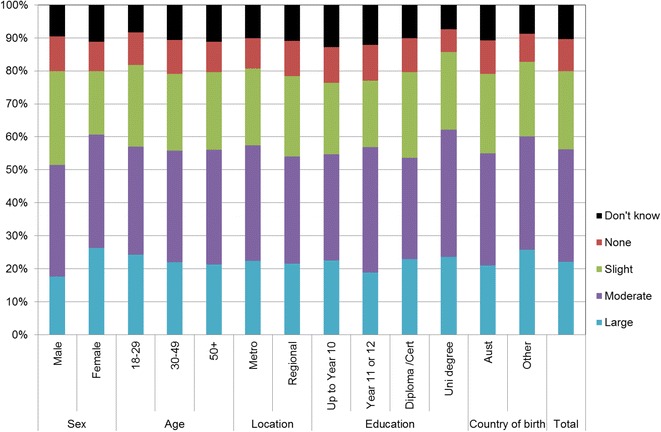
How much does drinking alcohol contribute to a person’s risk of getting cancer? Responses by respondent demographic characteristics

**Fig. 6:**
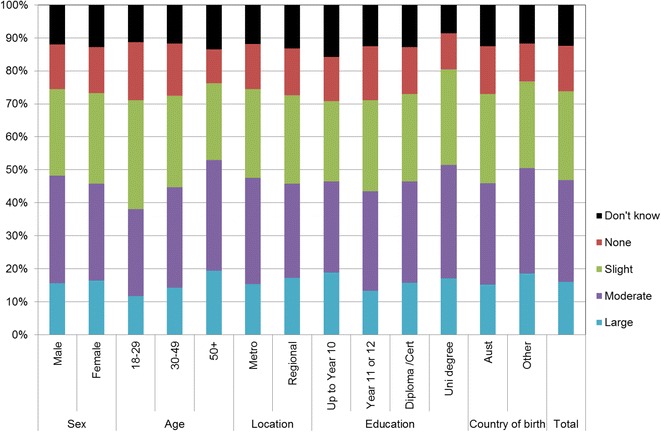
How much does not eating enough vegetables contribute to a person’s risk of getting cancer? Responses by respondent demographic characteristics

**Fig. 7:**
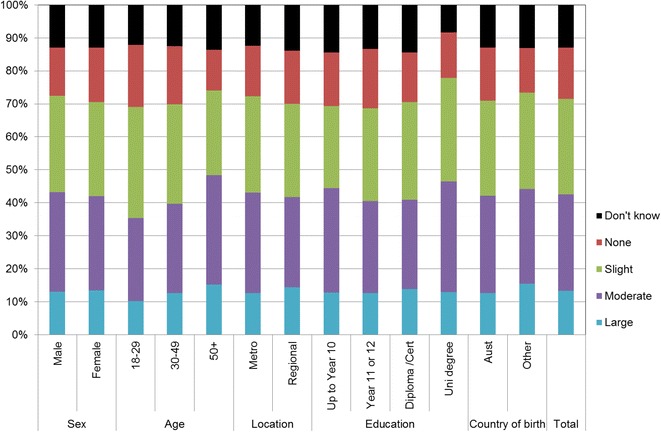
How much does not eating enough fruit contribute to a person’s risk of getting cancer? Responses by respondent demographic characteristics

**Fig. 8:**
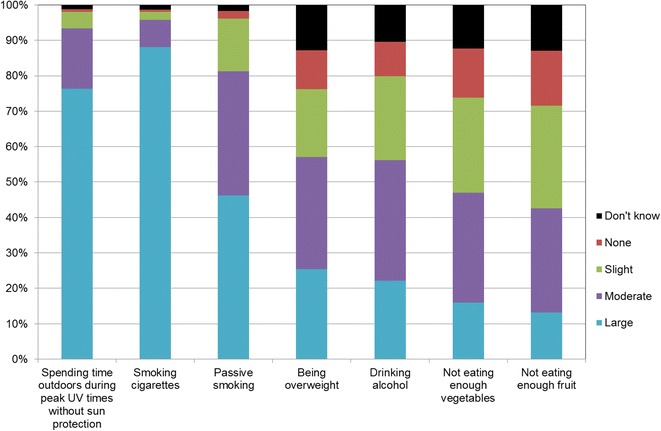
How much do each of the following things contribute to a person’s risk of getting cancer? Summary of responses for seven risk factors

Figures 1, 2, 3, 4, 5, 6, 7 show percentage distributions of respondent identification of each of the seven cancer risk factors as ‘Large’, ‘Moderate’, ‘Slight’, ‘None’ or ‘Don’t know’ by respondent demographic characteristics. Identification patterns show only minor differences across demographic characteristics. The main differences were between cancer risk factors, with greatest identification of smoking, UV exposure and passive smoking as ‘large’ or ‘moderate’ risks, and least identification of not eating enough fruit and vegetables (Fig. 8).

**Fig. 9:**
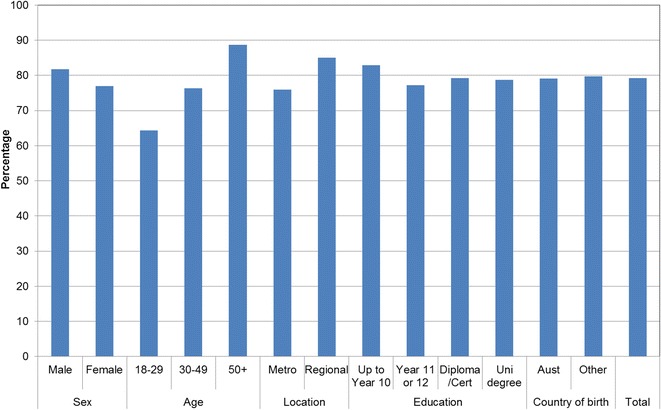
Percentage of respondents with ‘protective’ behaviour, by respondent demographic characteristics: sunsafe

**Fig. 10:**
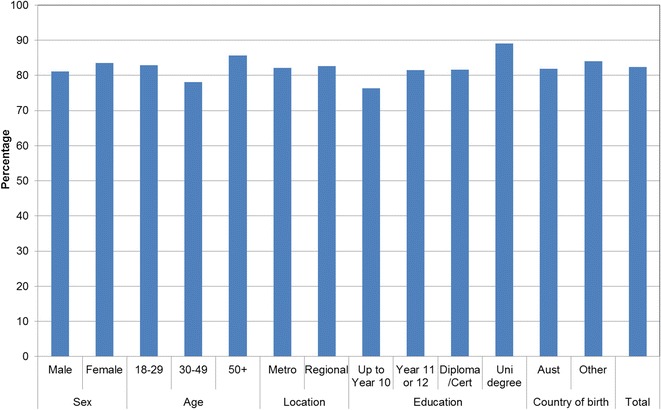
Percentage of respondents with ‘protective’ behaviour, by respondent demographic characteristics: non-smoker

**Fig. 11:**
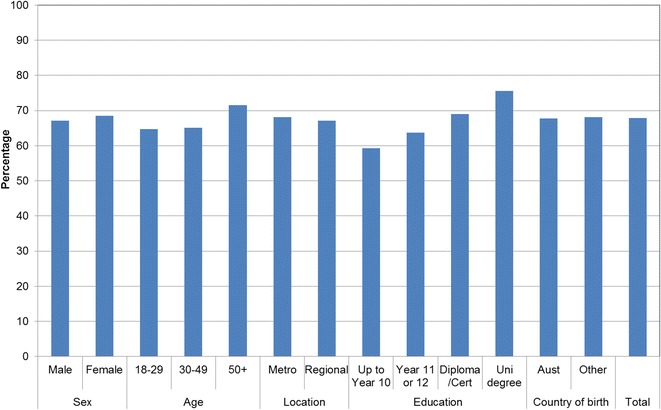
Percentage of respondents with ‘protective’ behaviour, by respondent demographic characteristics: avoid passive smoke

**Fig. 12:**
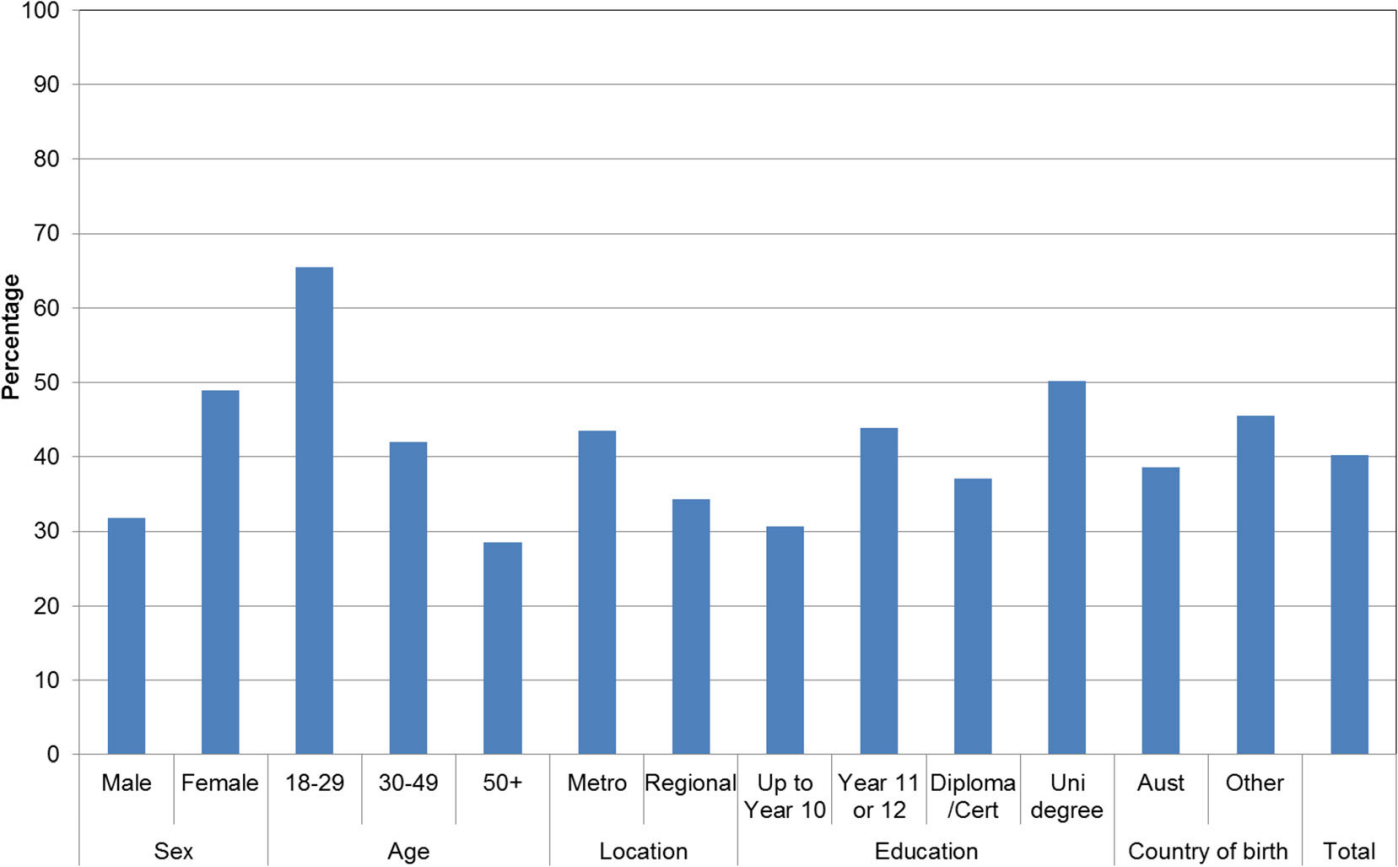
Percentage of respondents with ‘protective’ behaviour, by respondent demographic characteristics: healthy weight

**Fig. 13:**
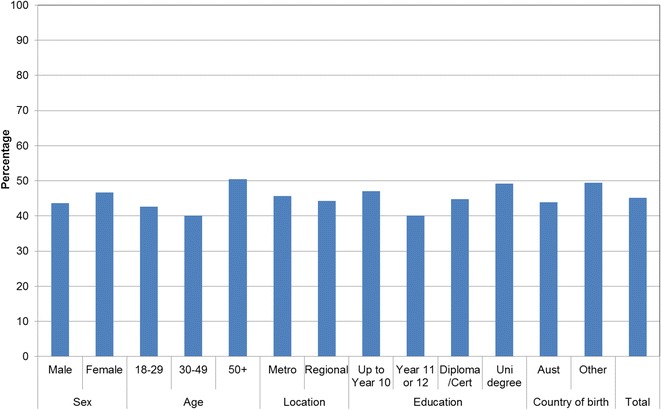
Percentage of respondents with ‘protective’ behaviour, by respondent demographic characteristics: lower-risk alcohol intake

**Fig. 14:**
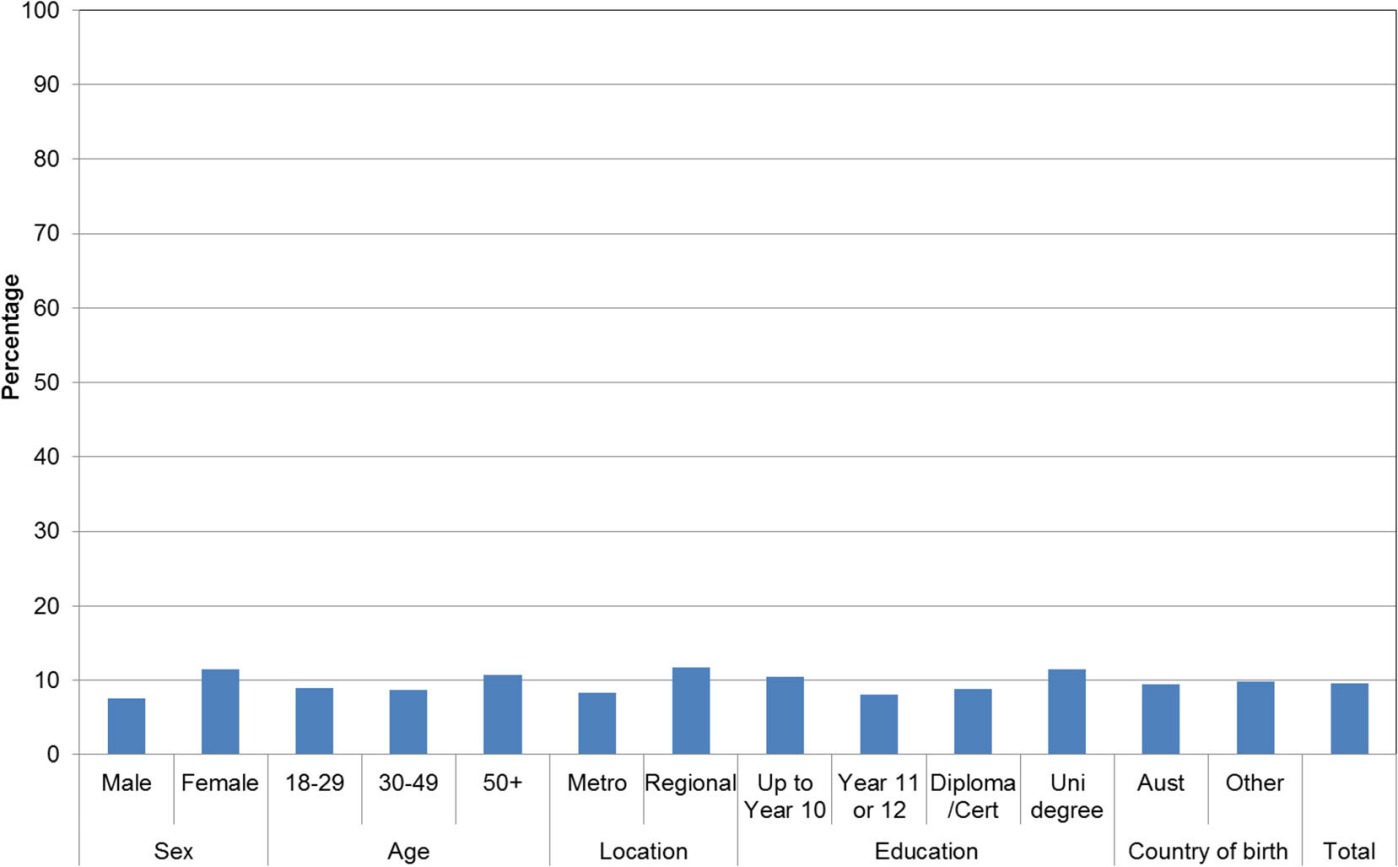
Percentage of respondents with ‘protective’ behaviour, by respondent demographic characteristics: five or more vegetable serves daily

**Fig. 15:**
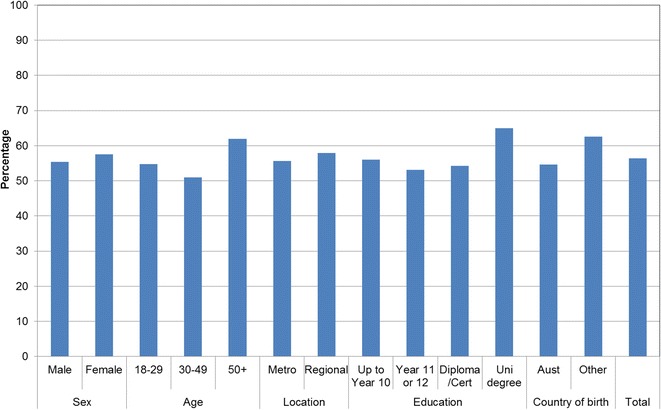
Percentage of respondents with ‘protective’ behaviour, by respondent demographic characteristics: two or more fruit serves daily

**Fig. 16:**
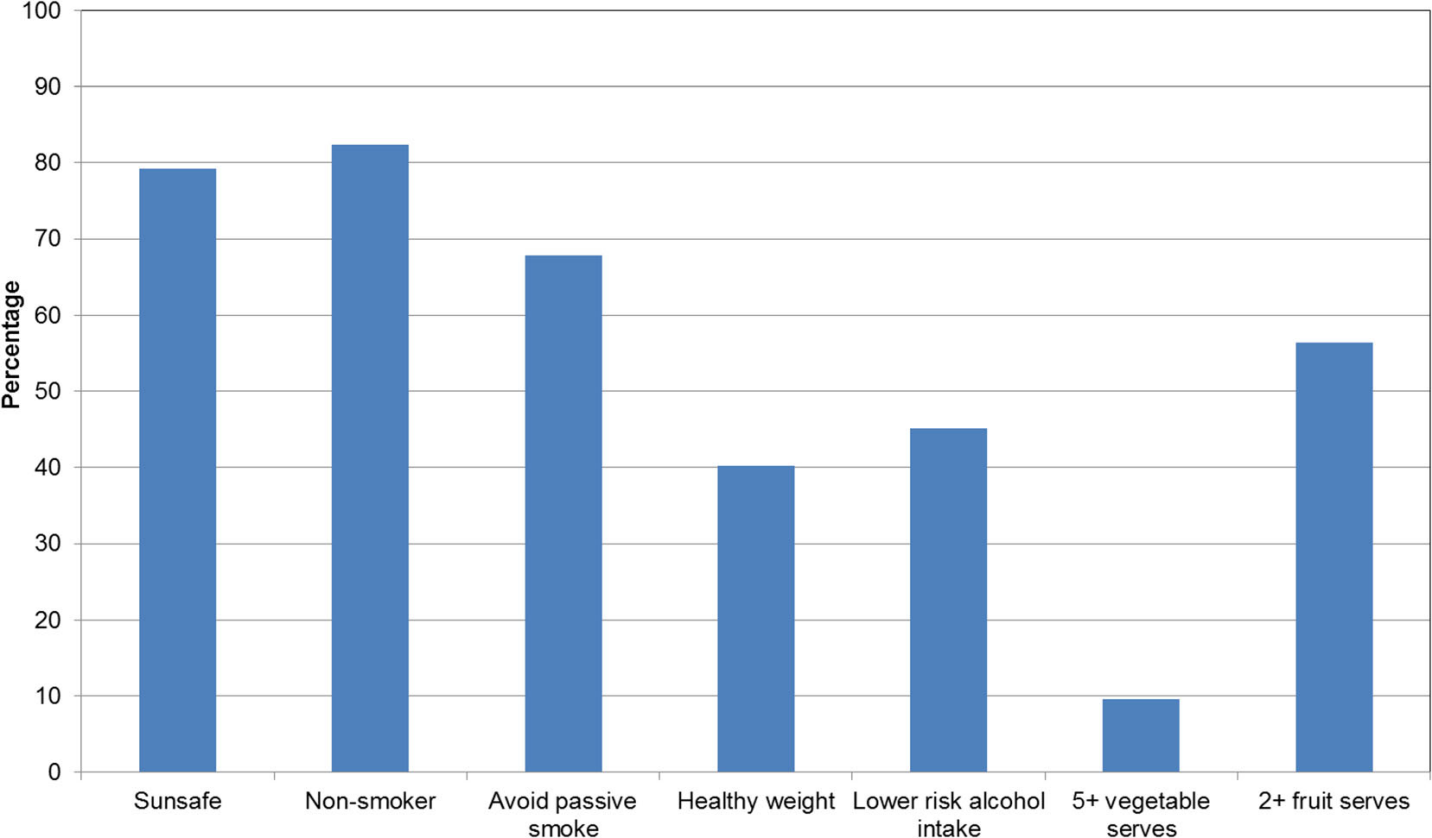
Percentage of respondents with ‘protective’ behaviour, by respondent demographic characteristics: summary of seven ‘protective’ behaviours

Figures 9, 10, 11, 12, 13, 14, 15, 16 show the percentage of respondents with ‘protective’ behaviours across the seven factors associated with cancer risk, by respondent demographic characteristics. Similar to the pattern seen with cancer risk factors, differences were greatest between behaviours than across demographic characteristics. Respondents were most likely to report sun protective behaviour (Fig. 9) and to be a non-smoker (Fig. 10), and least likely to consume the recommended daily intake of five or more vegetable serves (Fig. 14).

The relationship between respondent characteristics (sex, age, location, education and country of birth) and odds of identifying each cancer risk factor as ‘Moderate/large’ as opposed to ‘None/slight’ are shown in Table 2, with a logistic-regression model for each of the seven risk factors. Odds ratios are shown in the first column of each model, and *p*-values in the second column, with *p*-values of less than 0.05 considered to be significant. Significant values are shaded.

**Table 2:**
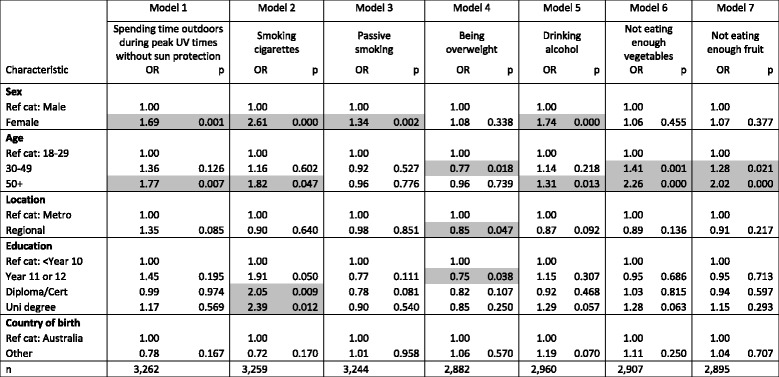
Logistic regressions

Identification of seven cancer risk factors. How much do each of the following things contribute to a person’s risk of getting cancer? Odds ratios of ‘Moderate/large’ response vs ‘None/Slight’ response (‘Don’t know’ excluded) by respondent characteristics

*OR* Odd ratio, *p p*-value, *Ref cat* Reference category, *n* sample number. *p*-values of less than 0.05 are shaded

Controlling for other factors, women were more likely than men to identify UV exposure, smoking, passive smoking, and drinking alcohol as moderate/large cancer risk factors. In comparison to respondents aged 18–29 years, those aged 50 years and over were more likely to identify five of the seven items as moderate/large risk factors. Those aged 30–49 years were less likely than those aged 18–29 years to identify being overweight as a moderate or large risk factor, but more likely to identify not eating enough vegetables and not eating enough fruit. Those living in regional areas of NSW were less likely than those resident in metropolitan area (Sydney) to identify being overweight as a moderate/large cancer risk factor. Those with post-school education were more likely than those with a Year 10 education or less to identify smoking as a moderate/large cancer risk factor, while those educated to Year 11 or 12 were less likely to identify being overweight. No significant results were found by country of birth.

Table 3 displays logistic regressions with respondent characteristics (sex, age, location, education and country of birth) as independent variables, and one of seven ‘protective’ behaviours—in relation to cancer risk factors—forming the dependent variable in each of the seven models. Female respondents were more likely than their male counterparts to practise all the ‘protective’ behaviours, except for being ‘sunsafe’. Respondents aged 30–49 years were more likely than those under 30 years to practise sunsafe behaviour, but less likely to be within the healthy weight range. Respondents aged 50 years and over were more likely than those aged 18–29 years to practise sunsafe behaviour, to be non-smokers and to avoid passive smoke, to have lower-risk alcohol intake and to eat two or more fruit serves daily. However they were also more likely to be overweight.

**Table 3:**
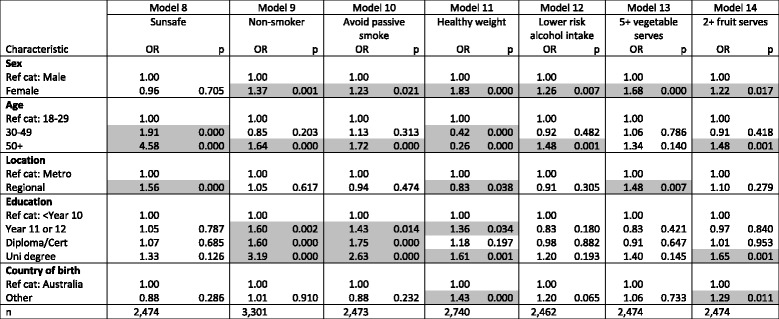
Logistic regressions

‘Protective’ behaviours by respondent characteristics

*OR* Odd ratio, *p p*-value, *Ref cat* Reference category, *n* sample number. *p*-values of less than 0.05 are shaded

Those living in regional areas of New South Wales were more likely to practise sunsafe behaviour and to eat five or more daily vegetable servings, but less likely to be within the healthy weight range. Respondents with post-Year 10 education were more likely to be non-smokers and to avoid passive smoke, those with Year 11 or 12 or a university degree were more likely to be within the healthy weight range, and those with a university degree were more likely to consume two or more servings of fruit daily. Respondents born outside Australia were more likely to be within the healthy weight range and to eat two or more daily fruit serves.

Table 4 shows relationships between identification of cancer risk factors as moderate/large and associated behaviours, controlling for respondent demographic characteristics. While women, older respondents and more educated respondents were more likely to report ‘protective’ behaviours related to cancer risk factors, the most important correlate of ‘protective’ behaviour across the seven items was found to be identification of the associated risk factor as moderate/large. Those who identify UV exposure as a ‘moderate’ or ‘large’ risk factor were more likely to report they did not try to get a tan nor did they use a solarium over summer (‘Sunsafe’) (Model 15). Identification of cigarette smoking as a moderate/large risk factor is correlated with being a non-smoker (Model 16). Those who report passive smoking as a ‘moderate’ or ‘large’ cancer risk factor were more likely to avoid exposure to others’ cigarette smoke (Model 17) even when controlling for smoker status (Model 18). Identification of alcohol as a moderate/large risk factor was associated with lower-risk drinking patterns (Model 20). Identification of not eating enough fruit as a moderate/large risk factor was associated with a higher probability of eating two or more serves of fruit daily (Model 22).

**Table 4:**
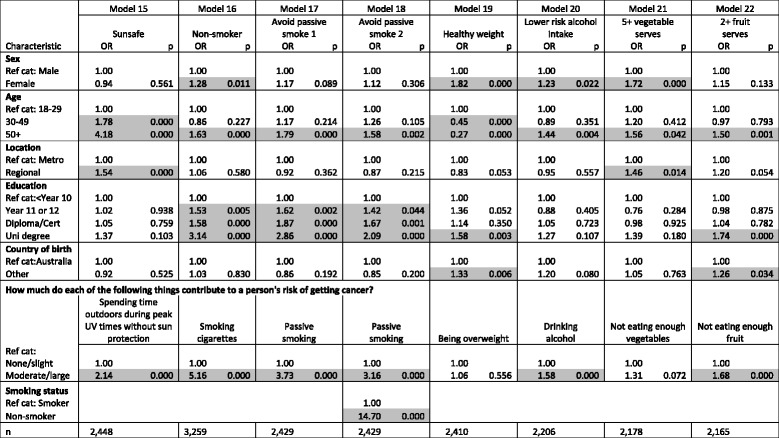
Logistic regressions

‘Protective’ behaviours by respondent characteristics and identification of related cancer risk factors

*OR* Odd ratio, *p p*-value, *Ref cat* Reference category, *n* sample number. *p*-values of less than 0.05 are shaded

Risk-factor identification as moderate/large was the greatest correlate for three of the seven ‘protective’ behaviours (‘Non-smoker’, ‘Avoid passive smoke’, and ‘Lower-risk alcohol intake’) and was significant for two others (‘Sunsafe’ and ‘2+ fruit serves). It was not a significant correlate for ‘Healthy weight’ nor ‘5+ vegetable serves’.

We ran other logistic-regression models to test whether identification of a cancer risk factor remained correlated with its associated ‘protective’ behaviour even when controlling for identification of other risk factors. We found that only identification of UV exposure was associated with sun protective behaviour; identification of none of the other six cancer risk factors was associated with sun protective behaviour. Similarly, only identification of drinking alcohol as a risk factor was associated with lower-risk alcohol intake. Identification of both smoking cigarettes and passive smoking as risk factors were associated with being a non-smoker and avoiding passive smoking, but not any other identifications. Broadly speaking, this indicates that behaviour and beliefs about risk and are specific to those single domains.

No risk factor identifications were correlated to being a healthy weight nor with consuming five or more vegetable serves daily. In contrast, identification of passive smoking, being overweight, drinking alcohol, not eating enough vegetables, and not eating enough fruit were all significantly associated with eating two or more serves of fruit daily.

## Discussion

This is one of the first studies to use multivariate analysis to examine the association between awareness of cancer risk factors and health behaviours across seven lifestyle-modifiable areas (sun exposure, smoking, passive smoking, healthy weight, alcohol, vegetable consumption, and fruit consumption). Increasing age was a significant predictor of all cancer protective behaviours and a range of demographic characteristics were associated with awareness and behaviours related to other cancer risk factors. After controlling for demographic factors, except for healthy weight and vegetable consumption, awareness of cancer risk was a significant predictor of the associated cancer protective behaviour.

The lack of correlation for healthy weight may have been because of the complexity of contributors to maintaining a healthy weight, and lack of self-awareness of weight status [20, 21]. The lack of correlation for ‘5+ vegetable serves’ may have been because so few respondents consumed this number of serves daily—less than 10%. When Model 21 was rerun with the dependent variable as ‘3+ vegetable serves’ (some health bodies recommend 5 serves daily of vegetables and fruit together), identification of ‘Not eating enough vegetables’ as a cancer risk factor became a significant correlate, with an odds ratio of 1.33 and a *p*-value of 0.001 (not shown).

### Awareness is important

Cancer prevention activities require a range of complementary approaches. There are a plethora of health promotion models and theories that guide many successful public health campaigns, and risk factor awareness is known to be important in influencing attitudes and intentions [22, 23]. Participants in this study reported low levels of awareness of four leading cancer risk factors: being overweight or obese, drinking alcohol, not eating enough vegetables, and not eating enough fruit (Figs. 4, 5, 6, 7). Historically, the largest and most significant cancer prevention campaigns in Australia have focused on UV exposure, and tobacco and related smoke exposure. These have resulted in significant improvements in community knowledge [8, 9]. However, community understanding of other risk factors remains poor [5–8, 24]. Cancer survivors and community population studies consistently report relatively high levels of awareness of the link between cancer and hereditary and environmental risks, but much lower levels of understanding about the role of lifestyle factors [5–7]. Social marketing campaigns are an effective approach in addressing such knowledge gaps and misconceptions about cancer risk factors [11]. Social marketing can change understanding and attitudes, and reframe social norms, but by itself does not change behaviour easily.

### Audience segmentation

Our findings suggest a role for audience segmentation and whole-of-population approaches in social marketing campaigns for cancer prevention. The link between each risk factor and associated behaviour was consistently significant amongst participants aged 50 years or more but this was not the case for other groups. Women, older people and to a lesser extent those with higher education (see Table 2) were most likely to identify UV exposure and cigarette smoking as cancer risk factors. This suggests there is a role for targeted social marketing campaigns, particularly addressing the beliefs of younger people and men about these risk factors. In contrast, community awareness of the cancer risk associated with high body weight, low vegetable/fruit intake and higher risk alcohol consumer was significantly lower than awareness of UV exposure and smoking cigarettes (see Table 2). There is a role for traditional population social marketing campaigns to enhance the awareness of these as cancer risk factors.

### Limitations

Our study had limitations. The survey was only conducted in one Australian state, and out of 30,179 initial email invitations, 10.9% of invitees completed the survey. While this may imply a bias, previous research has shown that response bias is minimised when considering correlation between variables in multivariate models, [25] as is the case in this study.

Study participants self-reported their behaviours. Thus there is the possibility of social desirability bias; that is, that respondents over-reported the positive behaviours measured. We also do not know what people had in mind when they identified risks as none, slight, moderate or large. We did not measure physical activity risk or behaviours, nor ask about dietary fibre and red and processed meat as other factors associated with cancer risk. Further study including these data would enhance our understanding of the relationship between these factors and healthy weight and nutrition behaviours.

## Conclusions

This study shows that there is high community awareness of UV exposure, smoking, and passive smoking as cancer risk factors. There is lower awareness of the link between cancer and being overweight, alcohol intake, and low fruit and vegetable consumption. Males and younger respondents are less likely to practise the behaviours that help protect against cancer. Since there is a strong correlation between awareness of cancer risk factors and practising the associated protective behaviours, these findings suggest a role for both audience segmentation and whole-of-population approaches in cancer-prevention social marketing campaigns. Targeted campaigns can address beliefs of younger people and men about cancer risk factors. Traditional population campaigns can enhance awareness of being overweight, alcohol consumption, and poor vegetable and fruit intake as cancer risk factors.

## Additional file

Additional file 1: 2013 Cancer Prevention Survey instrument. 2013 Cancer Council NSW Cancer Prevention Survey instrument. Extract of survey questions from the 2013 Cancer Prevention Survey instrument. This survey was conducted by Cancer Council New South Wales. (PDF 413 kb)

## Abbreviations

AUDIT-C: Alcohol use disorders—identification test—consumption
BMI: Body mass index
NSW: New South Wales
UV: Ultraviolet radiation
