# Supplementary material for: Identification of cancer risk and associated behaviour: implications for social marketing campaigns for cancer prevention
Source: BMC Cancer. 2017 Aug 17;17:550. doi: 10.1186/s12885-017-3540-x (PMC5561559; doi:10.1186/s12885-017-3540-x)
Supplement: Additional file 1: — 2013 Cancer Prevention Survey instrument. 2013 Cancer Council NSW Cancer Prevention Survey instrument. Extract of survey questions from the 2013 Cancer Prevention Survey instrument. This survey was conducted by Cancer Council New South Wales. (PDF 413 kb) [file 12885_2017_3540_MOESM1_ESM.pdf]

Cancer Council NSW Survey 2013

Extract of survey questions for *Identification of cancer risk and associated behaviour: implications for social marketing campaigns for cancer prevention*

Screening and demographics

S1 Where do you live?

Sydney and suburbs	1 - NOTE QUOTAS
Other New South Wales	2- NOTE QUOTAS
Melbourne and suburbs	TERMINATE
Other Victoria	TERMINATE
Brisbane and suburbs	TERMINATE
Other Queensland	TERMINATE
Perth and suburbs	TERMINATE
Other Western Australia	TERMINATE
Adelaide and suburbs	TERMINATE
Other South Australia	TERMINATE
Tasmania	TERMINATE
ACT	TERMINATE
Northern Territory	TERMINATE

S2 What is your age?

[ENTER TWO DIGITS. NOTE QUOTAS BASED ON FOLLOWING TABLE.]

Less than 18 years	TERMINATE
18-19 years	1- NOTE QUOTAS
20-29 years	2 - NOTE QUOTAS
30-39 years	3 - NOTE QUOTAS
40-49 years	4 - NOTE QUOTAS
50-59 years	5 - NOTE QUOTAS
60-69	6 - NOTE QUOTAS

70-79	7 - NOTE QUOTAS
80 or over	8 - NOTE QUOTAS

S3 Are you …[SINGLE RESPONSE]

MALE?	1 - NOTE QUOTAS
FEMALE?	2 - NOTE QUOTAS

S4 The highest level of education you have completed or are undertaking? [SINGLE RESPONSE]

Year 9 or below	1 - NOTE QUOTAS
Year 10	2 - NOTE QUOTAS
Year 11 OR 12	3 - NOTE QUOTAS
A diploma or certificate from a college or TAFE, including an apprenticeship	4 - NOTE QUOTAS
A degree from a university (including postgraduate degrees and diplomas)	5 – NOTE QUOTAS
Don't know	6 - TERMINATE

S5 Have you ever been diagnosed with any type of cancer by a doctor?

YES	1 – GO TO S6
NO	0 – GO TO S8
Prefer not to say	CONTINUE, BUT TERMINATE AFTER ASKING S9

S6 [IF YES AT S5] Are you currently undergoing medical treatment for your cancer?

Yes	1 – GO TO S7
No	0 – GO TO S8
Prefer not to say	CONTINUE, BUT TERMINATE AFTER ASKING S9

S7 [IF YES AT S6] What type of cancer have you been diagnosed with? [ACCEPT MULTIPLES]

Melanoma	CONTINUE, BUT TERMINATE AFTER ASKING S9
A skin cancer other than melanoma	GO TO S8
Another type of cancer	CONTINUE, BUT TERMINATE AFTER ASKING S9
I'm not sure	CONTINUE, BUT TERMINATE AFTER ASKING S9
Prefer not to say	CONTINUE, BUT TERMINATE AFTER ASKING S9

S8 Do you or anyone in your immediate family work in any of the following areas? [ACCCEPT MULITPLE RESPONSES]

Advertising	1 - TERMINATE
A business whose main purpose is to make or sell alcohol	2 - TERMINATE
A business whose main purpose is to make or sell tobacco	3 - TERMINATE
None of these	4 - GO TO S9

Topic 1: Knowledge of key cancer risk factor behaviours and their broader health consequences

1.6 How much do each of the following things contribute to a person's risk of getting cancer?

	Increase in cancer risk				
	None	Slight	Moderate	Large	I don't know
Passive smoking	0	1	2	3	99
Being overweight	0	1	2	3	99
Not eating enough fruit	0	1	2	3	99
Not eating enough vegetables	0	1	2	3	99

Drinking alcohol	0	1	2	3	99
Smoking cigarettes	0	1	2	3	99
Spending time outdoors during peak UV times without sun protection	0	1	2	3	99

Topic 2: Sun protection

2.2 Which of the following things have you done this summer?

	YES	NO	DON'T KNOW
Tried to get a tan from the sun	1	0	99
Used a solarium	1	0	99

Topic 3: Tobacco control

3.1 Which of the following best describes your smoking status? This includes cigarettes, cigars and pipes.

I smoke daily	1
I smoke occasionally	2
I don't smoke now, but I used to	3
I've tried it a few times, but never smoked regularly	4
I've never smoked	5

3.3 To what extent do you agree or disagree with these statements?

	Strongly disagree	Disagree	Neither agree nor disagree	Agree	Strongly agree	Don't know
I try to avoid places where I may be exposed to other people's cigarette smoke	1	2	3	4	5	99

Topic 4: Nutrition Component

Fruit

Individual Fruit Consumption

4.1 If a serve of fruit is equal to one medium piece or two small pieces of fruit, or one cup of diced fruit, how many SERVES of fruit do you eat each day, on average? (You may provide your answer to one decimal point.)

[RECORD NUMBER: 0 – 20. ALLOW ANSWER TO ONE DECIMAL POINT.]

Vegetables

Individual Vegetable Consumption

4.7. **If a serve of vegetables is equal to half a cup of cooked vegetables, one medium potato or one cup of salad, how many SERVES of vegetables do you eat each day, on average? (You may provide your answer to one decimal point.)**

[RECORD NUMBER: 0 – 20. ALLOW ANSWER TO ONE DECIMAL POINT.]

Topic 5: Alcohol

5.1. I have a drink containing alcohol …

4 OR MORE TIMES A WEEK	5
2 TO 3 TIMES A WEEK	4
2 TO 4 TIMES A MONTH	3
MONTHLY	2
LESS OFTEN THAN MONTHLY	1
NEVER	0
I DON'T KNOW	99

5.2 On a day when I am drinking, I usually have [BOX FOR PARTICIPANT TO ENTER NUMBER, MINIMUM 0, UPPER LIMIT OF 50.] standard drinks.

Please refer to the following chart which provides guidance on standard drinks.
[Standard drink chart provided]

5.3 How often do you have five or more STANDARD DRINKS on one occasion?

4 OR MORE TIMES A WEEK	5
2 TO 3 TIMES A WEEK	4

2 TO 4 TIMES A MONTH	3
MONTHLY	2
LESS OFTEN THAN MONTHLY	1
NEVER	0
I DON'T KNOW	99

D3 Where were you born? [SINGLE RESPONSE]

Australia	1 - CONTINUE
China	2 - CONTINUE
Germany	3 – CONTINUE
Greece	4 – CONTINUE
India	5 – CONTINUE
Italy	6 – CONTINUE
New Zealand	7 – CONTINUE
Netherlands	8 – CONTINUE
Philippines	9 – CONTINUE
UK, Channel Islands or Isle of Man	10 – CONTINUE
Vietnam	11 - CONTINUE
Other	12 - CONTINUE

D6 **How tall are you, without shoes on?** You may answer in centimetres, or in feet and inches.

1. ____ Centimetres

2. ____ Feet ____ Inches

98. I don't know

99. I prefer not to say

D7 **What do you weigh?** You may answer in kilograms, or in stone and pounds.

1. ____ Kilograms

2. ____ Stones ____Pounds

98. I don't know

99. I prefer not to say
